# Book Reviews

**Published:** 1891-07

**Authors:** 


### Book Reviews.

---

Transactions of the American Association of Obstetricians and Gynecologists. Vol. III., for the year 1890. Standard 8vo, pp, xliii.—385. Illustrated with nineteen plates, two charts, and forty-six engravings. Philadelphia: William J. Dornan. 1891.

The third volume of the transactions of this active and progressive association is fully up to the high standard of its predecessors. A glance at the table of contents will indicate the scope of the work performed at its Philadelphia meeting, and is as follows:

The President's Annual Address, by Edward E. Montgomery, M. D., Philadelphia; Some Facts Relating to the Diagnosis and Treatment of Placenta Previa, by James P. Boyd, M. D., Albany; Adherent Placenta: its Causes and Management, by Augustus P. Clarke, M. D., Cambridge; The Relation of Albuminuria to Puerperal Eclampsia, by William S. Gardner, M. D., Baltimore; Tincture of Iodine in Hyperemesis Gravidarum, by Llewellyn Eliot, M. D., Washington; The Practical Teaching of Obstetrics in the United States, by George H. Rohé, M. D., Baltimore; The Axis-traction Forceps: its Place in Obstetrics, by Joseph Hoffman, M. D., Philadelphia; The Vectis: a Useful, but Forgotten Instrument, by William Wotkyns Seymour, M. D., Troy; Shortening the Round Ligaments of the Uterus (Alexander's Method), by Joseph H. Branham, M. D., Baltimore; Ovarian and Ligamentous Cysts Co-existing in the Same Patient — Operation — Death from Shock, by William Warren Potter, M. D., Buffalo; The Surgical Conception of Peritonitis, by Joseph Price, M. D., Philadelphia; Some of the Difficulties met with in Abdominal Surgery, as Illustrated by Cases from Personal Records, by A. Vander Veer, M. D., Albany; Personal Experiences with Gall-stones and the Operation for their Relief, by William Wotkyns Seymour, M. D., Troy; The Elastic Ligature in Extra-peritoneal Treatment of the Pedicle, by X. O. Werder, M. D., Pittsburgh; Report of a Case of Extirpation of a Calculus from the Ureter by Combined Abdominal-lumbar Section — Recovery, by Rufus B. Hall, M. D., Cincinnati; Report of a Case of Hydatid Cyst of the Broad Ligament, by Norman Bruce Carson, M. D., St. Louis; Report of Gynecological Cases Treated by Electricity, by Franklin Townsend, Jr., M. D., Albany; A Discussion of Vaginal Hysterectomy, with Observations on Eleven Cases with one Death, by Charles A. L. Reed, M. D., Cincinnati; Puerperal Fever, by Wm. H. Myers, Fort Wayne; A Large Group of Mixed Specimens, Illustrating the Principal Complications and Varieties of Pelvic and Abdominal Surgery, by Joseph Price, M. D., Philadelphia; Specimens of Tubal Gestation, presented by E. E. Montgomery, M. D., Philadelphia; A New Mode of Exit for an Ovarian Cyst, by George R. Dean, Spartanburg; Description of Photographic Plates of an Edematous Acardia, by James F. W. Ross, M. D., Toronto; Infantile Vulvar Hemorrhage, by Thomas E. McArdle, M. D., Washington; In What Class of Wounds Shall We Use Drainage? by Henry O. Marcy, M. D., Boston; The Spleen as a Factor in Gynecology: with Report of a Successful Case of Splenectomy for Dislocation into the True Pelvis, by Frank A Glasgow, M.D., St. Louis; Osteomalacia Acutissima of the Anterior Pelvic Wall in a Rhachitic Woman, Developing Three Months after Delivery—Castration—Recovery, by F. Winckel, M. D., Munich; Extra-uterine Pregnancy—Laparatomy—Extraction of a Dead Child—Recovery; and Case of Tubal Pregnancy: Death from Rupture of Tube and Internal Hemorrhage, by Julius Nicolaysen, M. D., Christiania; Use and Abuse of the Obstetrical Forceps, by Eugene Prosper Bernardy, M. D., Philadelphia; Some of the Causes of Pelvic Disease, by Mordecai Price, M. D., Philadelphia; Chronic Purulent Endometritis, by John Chase Sexton, M. D., Rushville; Three Cases of Endometritis Treated with Chloride of Zinc Pencils, by A. Cordes, M. D., Geneva, Switzerland; Ectopic Gestation Two Etiological Inquiries, by W. A. Freund, M. D., Strassburg; The Etiology of Menstruation, by Milo Buel Ward, M. D., Topeka; Posterior Displacement of the Uterus — Remarks upon Pathology and Treatment, by Isaac S. Stone, M. D., Washington; Obscure Pelvic Inflammation Following Parturition, by Walter Coles, M. D., St. Louis; A Plea for Conservatism in the Treatment of Diseased Uterine Appendages, by Carlton C. Frederick, M. D., Buffalo; The Treatment of Fibroid Tumors of the Womb, by Donnel Hughes, M. D., Philadelphia; The Value of Exercise as a Therapeutic Means in the Treatment of the Pelvic Diseases of Women, by John Harvey Kellogg, M. D., Battle Creek; Malignant Degeneration in Dermoid Cysts, by George Erety Shoemaker, M. D., Philadelphia; The Operative Treatment of Recto-vaginal Fistula, by Dr. Max Sänger, Leipzig.

It will be observed that there is scarcely a subject that it is important to discuss at this period, in any of the departments covered by this Association, that is not here treated of, and the discussions were of the most spirited and instructive nature. Whoever would keep pace with the literature of obstetrics, gynecology, or abdominal surgery, will find it necessary to possess the annual volumes of this Association.

Book for Advertisers. Containing lists of the best newspapers in the United States and Canada. Together with a complete list of all the Class and Trade Journals. Being a compilation from the American Newspaper Directory, with Circulation Ratings and some Advertising Rates, together with a statement of the best way to place Newspaper Advertising. Small octavo, pp. 368. 178th edition. New York: George P. Rowell & Company. 1891.

Advertising has become a science, and Messrs. Geo. P. Rowell & Co. appear to have mastered it. It is difficult to see how so much information as this book contains could be condensed into so small a compass. The preparation of an advertisement to reach the eye of the party in greatest interest, and so to economize space as to bring its cost within the range of a proper limit to the advertiser's cash-box, is to be learned in this book,— and this is the sum of the whole subject. Daily, weekly, monthly and other periodicals and journals are all classified and rated in this work. Then there are statements made as to population, value of different newspapers and journals as advertising mediums, together with a vast amount of general and special information that should be in possession of every advertiser who would proceed intelligently to seek the eye of his would-be customer. No business man, journalist, advertising agent, or politician, can afford to deny himself the possession of this book, which can be obtained by sending its price, $1.00, to the publishers, No. 10 Spruce street, New York.

---

Wood's Medical and Surgical Monographs, consisting of original treatises and reproductions in English of books and monographs selected from the latest literature of foreign countries, with illustrations, etc. Published monthly, Vol. X., No. 1, April; No. 2, May; No. 3, June, 1891. New York: Wm. Wood & Co. $10.00. $1.00 per part.

The April number of the current volume contains the following titles: Treatment of Syphilis of the Nervous System, by Julius

Althaus, M. D.; Railway Injuries with Special Reference to those of the Back and Nervous System in their Medico-Legal and Clinical Aspects, by Herbert W. Page, M. A.; Causes and Prevention of Phthisis, by Arthur Ransome, M. D.

The May number contains: Differentiation in Rheumatic Diseases (so-called), by Hugh Lane, L. R. C. P.; Mental Affections of Childhood and Youth, and other papers, by J. Langdon Down, M. D.; Cure of the Morphia Habit, by Oscar Jennings, M. D.; Notes on the Examination of the Sputum, Vomit, Feces, and Urine, by Sidney Coupland, M. D.

The June number embraces the following subjects: Influenza Associated with Nervous and Mental Diseases, by Dr. VanDeventer; Technic of Ling's System of Manual Treatment, by Arvid Kellgren, M. D., Edin., illustrated with seventy-nine engravings; Antipyresis, by Prof. Arnaldo Cantani, Naples; Some Urinary Disorders Connected with the Bladder, Prostate, and Urethra, by Reginald Harrison, F. R. C. S., illustrated.

The variety of the titles here given and the renown of many of the authors, will ensure a large circulation of these numbers, which the publishers continue to present with the same liberality as to quality and price as heretofore.

DIABETES: ITS CAUSES, SYMPTOMS, AND TREATMENT. By CHARLES W. PURDY, M. D., Queen's University; Honorary Fellow Royal College of Physicians and Surgeons, Kingston; Member of the College of Physicians and Surgeons of Ontario, etc. No. 8 in the Physicians' and Students' Ready Reference Series. With Clinical Illustrations. Philadelphia and London: F. A. Davis. 1890. Price, $1.25.

The object of this work, to furnish the profession with its present status of knowledge on the subject of diabetes, is well fulfilled, and we regard it as a valuable *résumé* of this important subject for the use of the busy practitioner. Until the pathology of Diabetes is well established, and our therapeutic measures founded on a rational basis, the treatment must be empirical. The author does not aim to unravel this intricate problem, but gives the latest opinions in a style and clearness which makes his work a valuable addition to the medical library.

---

COSMETICS. A Treatise for Physicians and Pharmacists. By Dr. HEINRICH PASCHKIS, Docent of the University at Vienna. 8vo, pp. vi.—210. New York: William Wood & Company. 1891. Price, paper, $1.50.

The care of the skin and complexion are no longer trusted to the ignorant, nor are they governed by superstition or tradition. In this work we have the science of Cosmetics discoursed upon, as related to the care of the skin, hair and nails, and the mouth. Much importance is now attached to soaps, as it is well known that many times the use of impure or improper soap either causes skin diseases, or keeps them alive after they have appeared. Much information of value is given in this work in regard to soaps that every person should understand. The book also contains many new and useful formulæ, and will be found a valuable treatise.

All subscribers for the *American Druggist*, new orders or renewals, can receive the above-mentioned book FREE OF ALL CHARGE by settling their subscriptions, including arrears, direct with the publishers, as follows:

January subscribers who pay to the end of December, 1891; July subscribers who pay to the end of June, 1892.

Those desiring the book will please mention it when remitting.

